# Dengue Fever in Italy: The “Eternal Return” of an Emerging Arboviral Disease

**DOI:** 10.3390/tropicalmed7010010

**Published:** 2022-01-13

**Authors:** Matteo Riccò, Simona Peruzzi, Federica Balzarini, Alessandro Zaniboni, Silvia Ranzieri

**Affiliations:** 1 Servizio di Prevenzione e Sicurezza Negli Ambienti di Lavoro (SPSAL), Local Health Unit of Reggio Emilia, AUSL–IRCCS di Reggio Emilia, Via Amendola n.2, I-42122 Reggio Emilia, Italy; 2 Laboratorio Analisi Chimico Cliniche e Microbiologiche, Ospedale Civile di Guastalla, AUSL–IRCCS di Reggio Emilia, I-42016 Guastalla, Italy; simona.peruzzi@ausl.re.it; 3 Dipartimento P.A.A.P.S.S., Servizio Autorizzazione e Accreditamento, Agenzia di Tutela della Salute (ATS) di Bergamo, Via Galliccioli, 4, I-24121 Bergamo, Italy; federica.balzarini@gmail.com; 4 Department of Medicine and Surgery, University of Parma, Via Gramsci, 14, I-43126 Parma, Italy; alessandro.zaniboni@unipr.it (A.Z.); silvia.ranzieri@unipr.it (S.R.)

**Keywords:** spatio-temporal pattern, dengue, arthropod-borne virus, travel medicine

## Abstract

Enhanced surveillance for dengue virus (DENV) infections in Italy has been implemented since 2012, with annual reports from the National Health Institute. In this study, we summarize available evidence on the epidemiology of officially notified DENV infections from 2010–2021. In total, 1043 DENV infection cases were diagnosed, and most of them occurred in travelers, with only 11 autochthonous cases. The annual incidence rates of DENV infections peaked during 2019 with 0.277 cases per 100,000 (95% confidence interval [95% CI] 0.187–0.267), (age-adjusted incidence rate: 0.328, 95% CI 0.314–0.314). Cases of DENV were clustered during the summer months of July (11.4%), August (19.3%), and September (12.7%). The areas characterized by higher notification rates were north-western (29.0%), and mostly north-eastern Italy (41.3%). The risk for DENV infection in travelers increased in the time period 2015–2019 (risk ratio [RR] 1.808, 95% CI 1.594–2.051) and even during 2020–2021 (RR 1.771, 95% CI 1.238–2.543). Higher risk for DENV was additionally reported in male subjects compared with females subjects, and aged 25 to 44 years, and in individuals from northern and central Italy compared to southern regions and islands. In a multivariable Poisson regression model, the increased number of travelers per 100 inhabitants (incidence rate ratio [IRR] 1.065, 95% CI 1.036–1.096), the incidence in other countries (IRR 1.323, 95% CI 1.165–1.481), the share of individuals aged 25 to 44 years (IRR 1.622, 95% CI 1.338–1.968), and foreign-born residents (IRR 2.717, 95% CI 1.555–3.881), were identified as effectors of annual incidence. In summary, although the circulation of DENV remains clustered among travelers, enhanced surveillance is vital for the early detection of human cases and the prompt implementation of response measures.

## 1. Introduction

Dengue fever is a mosquito-borne tropical disease caused by a positive-sense, single-stranded RNA virus belonging to the family of *Flaviviridae* (dengue virus, DENV). DENV is subdivided in four distinct serotypes (DENV 1–4) that share a limited genetical identity (approximately 65–70% amino acid sequence similarity) [1,2,3]. A fifth serotype (DENV-5) was detected in Malaysia in 2007, but its significance remains unclear [3]. All serotypes in turn include several genotypes, i.e., a group of DENV isolates that have no more than 6% of nucleotide sequence divergence. Although all DENV serotypes can cause human disease, different serotypes or different genotypes within a serotype vary in terms of viral fitness, virulence, and epidemic potential [1,2]. Moreover, different lineages induce varied immune responses [4], with limited cross-lineage immunity.

Albeit up to 75% of incident cases of DENV infections usually remain unapparent and asymptomatic, they may also result in a wide range of clinical manifestations [3,5,6], ranging from a mild flu-like syndrome with fever, nausea, vomiting, rash, aches and pains (dengue Fever; DF) [7], to severe dengue hemorrhagic fever (DHF), and dengue shock syndrome (DSS), a syndrome characterized by an extensive capillary leak syndrome (possibly leading to shock), organ failure, and hemorrhagic manifestations, where mortality can be as high as 20% [4]. According to our current understanding, a main cause of DSS may be represented by partial immunity against the various DENV serotypes. More precisely, while one serotype elicits life-long immunity against its re-infections, it only provides a temporary and partial immunity against other serotypes, that may otherwise elicit an antibody-dependent enhancement (ADE) of natural infection. Heterotypic antibodies would then bind but not neutralize virions of the infecting DENV serotype, with resulting immune complexes that are recognized by cellular receptors that, in turn, facilitate virus entry and replication in target immune cells, with resulting vascular leakage and severe clinical features.

To date, DENV has been detected in more than 100 countries, with roughly half of the human population at risk [3]. Over the past decades, global incidence of dengue has dramatically increased, from 8.3 million in 1990, to 58.4 million in 2013, reaching around 100 million reported cases in 2019 [2,3,4,5,6,8]. Although the exact incidence of dengue is difficult to determine, the true number of annual infections may range from 284 to 528 million, with only one third to one fifth of cases being clinically symptomatic infections, including those that are undetected by reporting systems [4]. In other words, in less than a century DENV has evolved from a sporadic disease to a major public health problem with substantial social and economic effects [2,4]. This spectacular expansion has several reliable explanations. On the one hand, the rapid and uncontrolled urbanization in various geographic areas, particularly in south-eastern Asia, has drastically amplified both epidemic and endemic transmission cycles [2,3,5,6]. On the other hand, modern transport and enhanced globalization including rapid travel and trade have enabled the importation of DENV by overcoming natural barriers of travel time and geography, which had previously limited its expansion from endemic areas into non-endemic areas [2,3,5,9].

Interestingly, the European region has been mostly spared by the global spread of DENV. Despite the coexistence of permissive climatic factors and competent vectors (i.e., mosquitoes of the gender Aedes) [10,11], no large autochthonous outbreaks of DENV infections has yet occurred in the European area. In other words, to date dengue mostly remains a pathogen associated with international travel, being a relatively common cause of fever in travelers returning from South and Southeast Asia and Latin America [1,12,13]. Moreover, in Italy, despite the recent description of a small cluster of autochthonous DENV infections, dengue is usually acknowledged as a travelers’ disease. On the other hand, the aforementioned episode stress that, due to the presence of competent vectors, further importation and more sustained local transmissions in Italy are concrete possibilities [10,14,15]. As a consequence, interventions aimed to raise the awareness of medical professionals and health authorities may be particularly useful for the implementation of appropriate response measures, including blood safety, and preventive campaigns focusing on vector control and communication [7,9,15,16,17,18,19,20,21,22,23,24].

As a specific surveillance program has been implemented in Italy since 2012, our aim is to collect available evidence on the temporal and spatial patterns of dengue infections in Italy between 2010 and 2021, reporting appropriate insights from a country that has recently represented an example of extensive endemization of other arboviruses, such as West Nile Virus [25,26,27,28]. Furthermore, we will perform a comprehensive analysis on the possible influence of factors potentially involved in the epidemiology of dengue, focusing on sociodemographic factors (e.g., population, international travel, and number of incident cases in the nearby areas and in the rest of EU).

## 2. Materials and Methods

Settings: With a surface area of 301,340 km^2^ (116,350 sq mi), a total population of approximately 60 million inhabitants, and a population density of 201 people per square kilometer (520/sq mi), Italy is a densely but unevenly populated country in Southern Europe. Italy is administratively divided in 15 ordinary regions (i.e., Piedmont, Liguria, Lombardy, Veneto, Emilia-Romagna, Tuscany, Marche, Umbria, Latium, Abruzzo, Molise, Apulia, Campania, Basilicata, Calabria), 4 “special statute” regions (i.e., Aosta Valley, Friuli-Venezia-Giulia, Sicily, Sardinia), and 2 autonomous provinces (i.e., AP of Trento and Bolzano). On the other hand, Italy is usually divided in 5 large areas comparable in terms of overall population and more homogenous from an economic development point of view, that are represented by: north-western Italy (i.e., Aosta Valley, Piedmont, Liguria, Lombardy), north-eastern Italy (i.e., Veneto, AP of Trento and Bolzano, Friuli-Venezia-Giulia, Emilia Romagna), central Italy (Tuscany, Umbria, Marche, Latium); southern Italy (Abruzzo, Molise, Apulia, Basilicata, Campania, Calabria), and major islands (Sicily and Sardinia).

Data collection: Since 2012, a national surveillance plan for arboviruses was implemented, specifically focusing on Chikungunya, West Nile and Dengue Virus, with the publication of surveillance bulletins, with periodic updates. Periodic bulletins (available from: https://www.epicentro.iss.it/arbovirosi/bollettini; accessed on 26 October 2021) include the following data: total number of dengue infections reported during the surveillance year irrespective of their actual clinical features, month of reporting, age groups, sex, region of occurrence, whether the case was autochthonous or travel-related. According to the Italian case definition, the diagnosis of dengue occurs in subjects with suggestive clinical features (i.e., fever > 38°C during the past 7 days in a traveler who had returned within the previous 15 days from countries to which these viruses are endemic, absence of leukocytosis (leukocyte count <10,000 μL) [14,19,24], and absence of other obvious causes of fever), and a positive confirmatory test represented by:

(a) days 0 to 8 from the onset of clinical symptoms, a positive serum PCR, OR seroconversion test by commercially available IgM, OR identification of viral antigen;
(b) since the 9th day from the onset of symptoms, seroconversion test by commercially available IgM/IgG

As data on the age groups and gender of reported cases were inconsistently reported by periodic bulletins, information was complemented by high-quality and highly reliable data from the ECDC Surveillance Atlas of Infectious Diseases (https://atlas.ecdc.europa.eu/public/index.aspx; accessed on 26 October 2021), and by pooling available information from competent regional health authorities.

For each study year, data on the Italian population, in total, and for affected regions (i.e., regions where cases had been notified during the surveillance period) were retrieved from the Italian National Statistical Institute (ISTAT; http://demo.istat.it/; accessed on 26 October 2021). As dengue is historically a travel-related infectious disease, data on travel (i.e., number of travelers from Italy, number of travels performed by years, by large areas) were also collected from the open ISTAT platform “DATI” (http://dati.istat.it/, accessed on 26 October 2021).

Statistical analysis: We performed descriptive analysis of the surveillance data, i.e., geographical and temporal distribution of dengue cases, with their respective demographic characteristics (age and sex). We then analyzed the corresponding annual incidence rates between 2010 and 2021, both for Italian residents, and travelers. Seasonal trends were then assessed by normalizing the number of monthly reported cases by the maximum number of new cases, both in Italy and in other EU countries. Eventually, age adjusted incidence rates (ASR) for every Italian region were calculated for dengue assuming the European standard population as reference [29].

The relationship between the number of infections and the demographic factors was investigated through calculation of incident rate ratios (IRRs) with their correspondent 95% CI in a Poisson regression model that included as the outcome variable the yearly incident rates for dengue cases (assessed at regional level). The explanatory variables were represented by travel-related (i.e., number of travels; share of travels to high-risk areas of Asia and Central/South America) and demographic factors (i.e., number of dengue incident cases in the rest of EU, share of population aged 25–55 years).

All calculations were performed on R 4.0.3 [30] by means of packages epiR (v. 2.0.19), EpiReport (v 1.0.1), fmsb (0.7.0), plot3d (1.3), msm (1.6.8), and sandwich (3.0–0).

Ethical approval: No ethical approval was needed for this study, as no individual data were identifiable, and only aggregated data were analyzed and presented.

## 3. Results

### 3.1. Demographics

In total (Table 1), 1043 cases of dengue cases were notified in Italy between 2010 and September 2021 (average 86.9 cases, ranging from 4 to 185). The majority of reported cases occurred in individuals of male gender (57.0%), and more than half of them were aged between 25 and 44 years (51.6%). The number of reported cases increased in the five-year timeframe 2014–2019 compared to 2010–2019 (58.4% vs. 38.2%), with an average 79.6 cases/year vs. 121.8 cases/year, respectively. On the contrary, during the timeframe 2020–2021 only 36 incident cases were reported, including 11 (2.5%) with a documented autochthonous origin. The majority of cases were reported from northern Italian regions (29.0% from north-western Italy, and 41.3% from north-eastern Italy), while less than 1/3 of new diagnoses were reported from central and southern Italy, including the main islands of Sicily and Sardinia (25.9%, 2.5%, and 1.8%, respectively).

**Figure 1:**
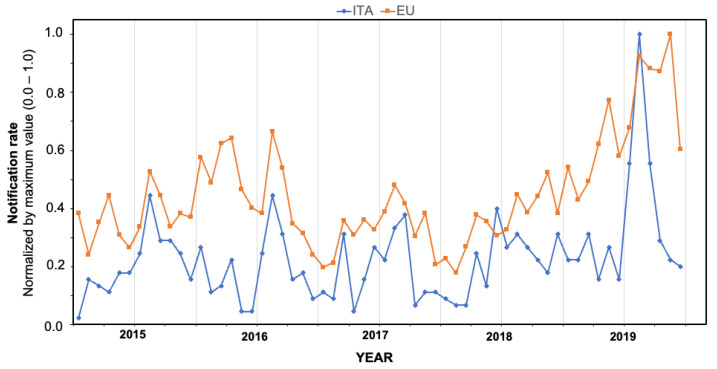
Comparison of Italian and European data for dengue in the time period 2015–2019, monthly incident cases, normalized by maximum value.

**Figure 2:**
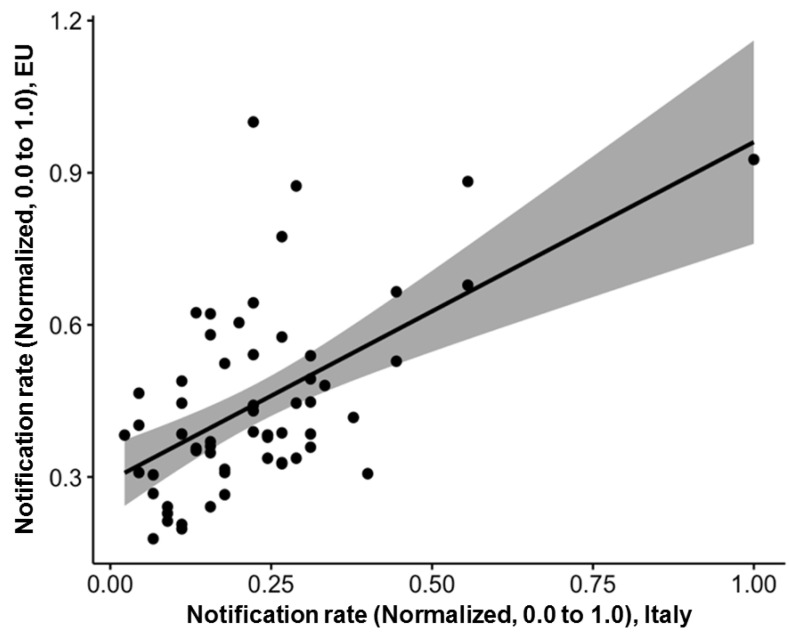
Correlation between notification rates in Italy and in the rest of European Union, reported as monthly rates normalized by maximum values. Notification rates per 100,000 persons and foreign-born residents per 100 people, calculated at regional level (Italy, 2010–2020) (R = 0.48, *p* < 0.001).

Unfortunately, data on the seasonal trend and the geographic region of importation of the reported cases were more fragmentarily available (respectively, for the time period January 2015–August 2021; and for the reporting years 2010, 2012–2016, 2018–2021, for a total of 648 and 446 cases). According to available data, cases were notified across all of the calendar year, but the majority of them occurred during the warm season, June to September (in total 57.9%), peaking in August (19.3%), followed by September (12.7%), July (11.5%), and June (7.4%). Overall, normalized occurrence of new diagnoses substantially mirrored that of European Union (Figure 1), being substantially well correlated (Figure 2; R = 0.48; *p* < 0.001).

Focusing on the suspected source of infection, the majority of cases had a previous sojourn in Asian countries, either southern and eastern Asia (26.9%) or Southeast Asia (25.1%), followed by Caribe (19.7%; for a total of 26.2% when considering all the Latin American countries as a whole), Africa and Middle East (6.7%).

### 3.2. Estimates for Notification Rates

As shown in Table 2, crude annual notification rates for dengue ranged from 0.011 cases per 100,000 (95% CI 0.001–0.015) in 2021 and 0.032 cases per 100,000 (95% CI 0.001–0.064) in 2020, to 0.227 per 100,000 (95% CI 0.187–0.267) in 2019, with a pooled estimate equal to 0.114 per 100,000 (95% CI 0.071 to 0.183). When notification rates were standardized according to the European Reference population, ASR ranged between 0.008 per 100,000 (95% CI 0.004–0.011) in 2021, and 0.328 (95% CI 0.314–0.341) for 2018, with a pooled estimate of 0.155 cases per 100,000 (95% CI 0.105–0.206) for the whole of the assessed timeframe.

On the contrary, rates for travelers ranged from a nadir of 0.325 cases per 100,000 travelers (95% CI 0.242–0.427) in 2010, to an apex of 1.514 (95% CI 1.305–1.746) in 2019. As no reliable data were still available regarding calendar year 2021, the pooled estimate was calculated for the time period 2010–2020, being equal to 0.796 per 100,000 (95% CI 0.599–1.057).

**Figure 3:**
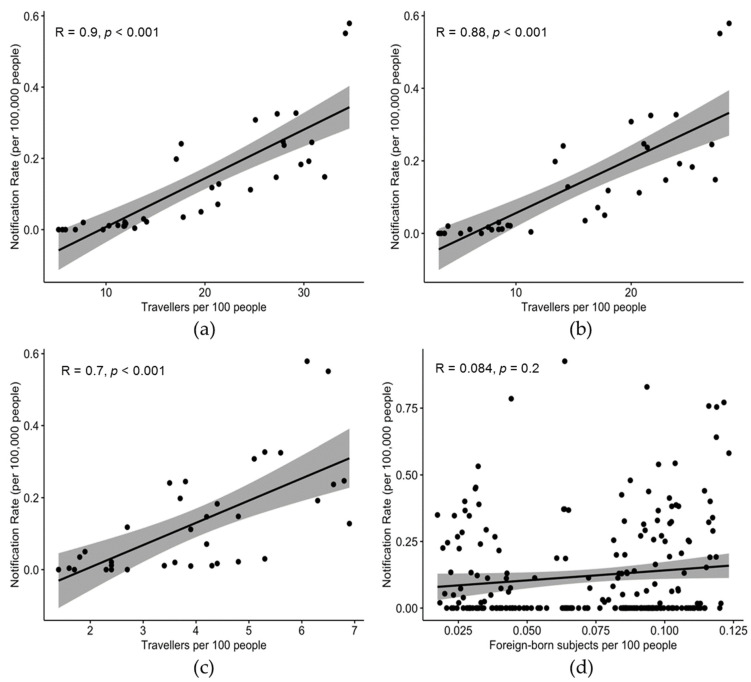
Correlation between notification rates (per 100,000 people) by year or reporting (2014–2020), and: the share of travelers per 100 people by geographic area (i.e., north-western, north-eastern, central, and southern Italy, and main islands), in general (**a**); including only non-work-related travel (**b**); including only work-related travel (**c**); the share of foreign-born subjects per 100 people by year and reporting region (**d**).

In fact, incidence rates were well correlated, not only when considering the entirety of travelers (R = 0.900, *p* < 0.001) (Figure 3a), but also when dichotomizing non-work-related and work-related travels (R = 0.700, *p* < 0.001; and R = 0.88, *p* < 0.001, respectively; Figure 3b,c).

The occurrence of new cases of dengue on a regional basis was quite heterogeneous during the entire assessed time period. The higher notification rates for dengue were from Emilia-Romagna region (0.375 per 100,000; 95% CI 0.257–0.492), followed by Veneto (0.317 per 100,000; 95% CI 0.245–0.388), and then the AP of Bolzano. On the contrary, not only were some regions only occasionally reporting new cases during the assessed time frame (i.e., Aosta Valley; Liguria, Abruzzo, Campania, Calabria and Sardinia), but two southern regions did not report any cases at all (i.e., Basilicata and Calabria). No significant correlation was found with the number of foreign-born residents per 100 people when calculated at a regional level (R = 0.084; *p* = 0.351). Detailed incidence rates by province during the assessed timeframe are provided in Table A1 (Appendix A).

### 3.3. Univariate Analysis

As shown in Table 3, female individuals exhibited a substantially reduced risk ratio (RR) for dengue infection (RR 0.704; 95% CI 0.622–0.796) compared to male individuals. Similarly, univariate analysis confirmed an increased risk for dengue infection in individuals aged between 25 and 44 years, as correspondent RR was substantially reduced in all other age groups. Focusing on the reporting region, we arbitrarily assumed residents from north-western Italy as the reference group, as this area includes the region of Lombardy, which is not only the most populated region of the whole country (i.e., 16.9% of the total Italian population, according to 2021 census) but is also characterized by the highest socioeconomic developmental indices [27,31]. A substantially increased occurrence was identified in subjects from north-eastern (RR 1.968, 95% CI 1.699– 2.280), and central Italy (RR 1.205, 95% CI 1.020–1.417), while starkly reduced estimates were calculated from southern Italy and main islands (RR 0.102, 95% CI 0.069–0.152; and RR 0.111, 95% CI 0.065–0.191).

Interestingly enough, when reporting years were taken into account, and assuming the 5-year timespan 2010–2014 as the reference category, the risk for dengue in residents was substantially increased for the time period 2015–2019 (RR 1.552, 95% CI 1.352–1.782), and conversely reduced for the time period 2020–2021 (RR 0.487, 95% CI 0.339–0.699). On the contrary, in travelers the risk of developing dengue was increased in both the timeframes of 2015–2019, and 2020–2021 (RR 1.808, 95% CI 1.594–2.051; and RR 1.771, 95% CI 1.238–2.534).

### 3.4. Multivariable Analysis

When notification data were included in a Poisson regression model such as: occurrence of new cases in the rest of EU-EEA; the population aged 25–44 years; the share of foreign-born residents, the number of travelers per 100 residents; the number of travels to South and Central America and Asia (including southeastern and South/East Asia as a whole) (all of them reported by region of origin), a distinctive pattern was identified. As shown in Table 4, the effective explanatory variables of new cases were identified in the share of travelers per 100 population irrespective of their geographic area of destination (+1.0%, IRR 1.065, 95% CI 1.036 to 1.096), the share of residents aged between 25 and 44 years (+1.0%, IRR 1.622, 95% CI 1.338 to 1.968), and foreign-born (+1.0%, IRR 2.717, 95% CI 1.555 to 3.881), and in the total number of incident cases in EU (+1000 cases/year; IRR 1.323, 95% CI 1.165 to 1.481) in the reporting year.

## 4. Discussion

During the last century, dengue has quite rapidly evolved from a marginal disease to a global public health threat, with considerable costs in both economic and social terms [2,3,5,6,8]. Although an increasing number of countries have evolved into high-risk areas for dengue, until recently western Europe had been substantially spared by the global pandemic. In fact, with the notable exception of the Portuguese island of Madeira, which in 2012 was involved in a large local outbreak (in total, 1080 confirmed cases), the European Centre for Disease Prevention and Control (ECDC) still considers DENV as not endemic in mainland EU/EEA, with the vast majority of all cases occurring in travelers infected outside the mainland EU/EEA [32]. Not coincidentally, our retrospective analysis of Italian National Reports 2010–2021 was able to retrieve a total of 1043 cases, but only 11 of them (1.1% of total cases; 2.5% of all cases with a reported analysis of the potential source) had a confirmed autochthonous spreading [14], while the remaining cases were associated with international travel, mostly from geographic areas with an established status of high-risk areas for dengue, namely South-East Asia, southern and eastern Asia, and Latin America. In this regard, during the very same timeframe assessed in this review, i.e., between 2010 and the travel ban and subsequent restrictions associated with SARS-CoV-2 pandemic, Italy had experienced a substantial and sustained resurgence of outbound travels, trips and holidays abroad (around 20.7% of 78,940 million trips with overnight stays in 2018), that in 2008 had experienced a historic drop associated with the economic crisis. Although the large majority of travels involved other EU countries, a sustained increase of travel to high-risk areas in Asia (from 553,149 in 2014 to 842,950 in 2020) and Latin America (from 135,689 to 239,590) has been reported [9,33].

In fact, our pooled estimates confirmed some previous reports from main Italian reference centers. For instance, Pagani et al. retrieved and analyzed the epidemiological and clinical features of imported dengue fever observed at Luigi Sacco Hospital’s Department of infectious diseases in Milan over a 16-year time period (2001–2016) including a total of 122 patients (106 probable and 16 proven diagnoses) [1]. Consistent with our estimates, the majority of them were young (median age: 35 years), and Italian-born individuals (91%), mostly returning from Asia (*n* = 67; 55%), and either Central America/Caribbean area (*n* = 27; 22%) or South America (*n* = 16; 13%). Similarly, Nicoletti et al. [34] studied 76 travelers or migrants from areas endemic to CHIKV/DENV, and 17.1% of them were positive for DENV. Persons who had visited Asia had a higher risk for DENV infection compared to those who had visited other areas (OR 8.36, 95% CI 1.58–81.73). Eventually, Pierro et al. reported on 83 serum samples from febrile travelers returning from dengue-endemic countries to Emilia Romagna, with 17 positive cases for dengue [35]. Focusing on the potential source of exposure, positive cases had recently visited either southeastern Asia (10/17), Caribbean region and/or Latin America (6 out of 17). Interestingly enough, our study suggests a substantial prevalence of cases in male subjects. This specific topic has been inconsistently reported across available reports [1,12,16,32], but could be explained as a consequence of several factors, including the differences in the use of health services, a different adherence to preventive measures in high-risk areas, but also a difference in gender roles particularly when dealing with travelers visiting friends and relatives, and for work-related travels, with subsequent differences in exposure risks [36].

As our review has substantially stressed the nature of dengue in Italy as a travel-related disease, additionally the seasonality of the reported cases mirrored that of travels in pre-SARS-CoV-2 pandemic era, with most cases occurring during the summer season. Not coincidentally, also in previous reports almost half (*n* = 52, 43%) of the patients were observed in August and September [1], i.e., the summer holiday season for most of Italian workers. As we were unable to systematically retrieve the cause of travels from reported cases, in our analyses we focused on a proxy represented by the number of foreign-born individuals from the reporting region. Travelers visiting friends or relatives are a well-known high-risk group for certain pathogens [21,23,37,38], including DENV. In this regard, previous Italian reports have quite consistently reported leisure trips as the main reason for the travels associated with DENV infection, and only a more marginal share of individuals had reportedly visited friends or relatives [15,16,17,21,23,34,38]. However, our estimates clearly identified the share of individuals with a migration background in the parent Italian region as a significant effector of the annual incidence of dengue. A possible explanation may be represented by a substantial correlation in-between the migratory phenomenon and the economic development of the reference Italian region [39,40,41]. North western and north eastern Italian regions represent the most developed and productive areas of the country, with one of the highest GDPs per capita in Europe. On the one hand, these regions have therefore become particularly attractive for migrant workers, with a higher share of individuals with a 1st or even 2nd generation migration background [40,42]. On the other hand, the higher purchasing power of residents from these areas increases the opportunities for traveling abroad, including high-risk areas from South America and Asia.

In other words, at least until a clear and sustained circulation of the pathogen occurs in continental Europe, our estimates suggest that the true main effectors of the occurrence of dengue in Italy should be identified in the global trend of the infection in high-risk areas, and in the accessibility of travelers to these areas [3,11,13,16,17,35,43,44]. First, we found a clear correlation of Italian incidence rates with EU-EEA estimates, where dengue has mostly remained a travel-associated infection [9,12,15,21,34,38]. Second, the crude risk for DENV infection remained particularly high in the pandemic years 2020–2021 despite the dramatic reduction of overseas travel, particularly to countries of eastern and south-eastern Asia, where the “zero-COVID strategy” has been largely implemented, and international travels have been either banned or severely restricted [45,46]. In fact, international estimates suggest that—in high-risk areas characterized by documented circulation of the pathogen, physical distancing and non pharmaceutic interventions (NPI) implemented to cope with SARS-CoV-2 did not have a noticeable impact on arboviruses such as DENV, as it was previously stressed for other mosquito-borne pathogens such as WNV [26,27].

Comfortable as these estimates may appear, it should be stressed that at least one of the main vectors for DENV, *Aedes albopictus*, is established in the southern and central parts of mainland EU [10,22,47,48], and climate change is accelerating its spread [3,49]. From this point of view, it should be stressed that large areas of Italy, particularly in the northern regions, should be acknowledged as high risk of endemization for all arthropod-borne pathogens [25,26,27,28]. The Po River valley, for example, is a humid, subtropical area with intensive rainfalls that occur across the entire calendar year. Summer average temperatures usually range between 22 and 25°C, but daily maximum temperatures may be higher than 35°C [31,50]. Combined with the milder winter temperatures associated with climate change, these conditions are quite favorable to the ecology of mosquitoes, including species potentially competent for DENV [51,52]. In fact, the potential impact of arthropod-borne pathogens in Italy is all but new: not only has malaria been endemic in the large areas of Italy (i.e., Po River Valley and Latium) since the late antiquity until the 1950s [53], but northern Italy has hosted in 2007 the first European outbreak of autochthonous cases of chikungunya [13], and Po River Valley has been acknowledged as a substantial hotspot for WNV [26,27].

Interestingly, a recent summary of ECDC on suspected autochthonous cases of dengue in mainland EU has identified several, independent foci in southern France (i.e., Departments of Hérault, Var, Alpes Maritime) [54]. In other words, dengue should still be acknowledged as an “alien” and sporadic pathogen on the edge of endemization, and on the very same path previously followed by other arboviruses such as WNV. In this regard, it should be stressed that while no vaccines against WNV are to date available, a vaccine for DENV (Dengvaxia^®^) has been approved by the U.S. Food and Drug Administration (FDA) in the United States for use in children aged 9 to 16 years with laboratory-confirmed previous dengue virus infection and residence in an area where dengue is endemic (where dengue occurs frequently or continuously) [55,56,57]. Moreover, in June 2021, the Advisory Committee on Immunization Practices (ACIP) recommended use of Dengvaxia^®^ to prevent dengue in children aged between 9 and 16 years [58]. To date, no recommendations have been issued for travelers. While in high-risk areas the massive vaccination campaigns may be cost-effective [59], available data hardly support the extensive vaccination against DENV in travelers [60]. In settings characterized by very limited autochthonous circulation, if any, and relatively few reported cases (in the peak years of 2019, 1.514 per 100,000 travelers, 95% CI 1.305–1.746), despite its reported efficacy, with a cost of around US Dollars 60–69 for completing the three-dose schedule, total costs would overwhelm potential sparing [56,57,61,62]. In fact, according to ISTAT estimates on travel between 2010–2019, an average of around 538,383 travelers/year to high-risk areas in Asia and 279,029 travelers/year to South America may be calculated. In a worst-case scenario where all travelers are required to complete their vaccination schedule, expenses faced by Italian National Health Service would range between EUR 49,044,720 and EUR 56,401,428 in order to avoid no more than 185 cases (i.e., the peak of crude incidence from 2019). Even assuming that all of them may have required advanced healthcare, correspondent efficacy of Dengvaxia^®^ in avoiding recipient hospitalizations ranges between 80.8% in adults and 56.1% in children [55,56,57,61]: as 93.7% of reported cases occurred in adults, if all individuals traveling to high-risk areas would receive a full vaccination schedule, assuming a scenario similar to 2019, vaccination would have spared around 146 cases, with a total cost of EUR 335,922 to EUR 386,311 for averted cases.

*Limits.* Despite their potential interest, our data are affected by significant limits. First, we have drawn our estimates from national bulletins, and therefore we were able to summarize and analyze only the information that was preventively reported and analyzed by national authorities [7,9,14,20,21,24]. Moreover, we lack significant data about the demographics of a significant portion of reported cases, with resulting uncertainties in eventual estimates. Moreover, significant information on the reported signs and symptoms were not available to our analysis. According to 2009 WHO case classification, DENV infection is classified in probable dengue, dengue without warning signs, dengue with warning signs and severe dengue, but such information is not provided by national bulletins [2]. Further analyses on available reports from reference centers are therefore required in order to improve our understanding of the clinical features of DENV infections in Italian travelers [1,15,16,18,20,21,35]. Second, national bulletins forcibly oversampled symptomatic cases, which represent only a fraction of total DENV infections [2,3,4,5,8]. Third, we are substantially deprived of information about the potential settings for DENV infection. DENV is transmitted in both urban (human transmission cycle) and forested areas (sylvatic transmission cycle). Both of these transmission cycles are different ecologically and evolutionarily [4], and according to recommendations of WHO, epidemic surveillance is essential to properly develop effective dengue prevention and control programs [63]. As DENV has shown the potential to develop autochthonous transmission, following the footsteps of other arthropod borne viruses such as WNV [26,27,64,65], appropriate characterization of cases remains the cornerstone for controlling the early emergence of DENV outbreaks, alongside the control of potential vectors [13,14,63].

Fourth, data on demographics and travel that we included in our models should be assumed as a proxy. Travelers visiting friends and relatives often undertake last-minute travel, may stay for longer periods than other groups of visitors, live in close proximity to local populations, and may therefore be at a higher risk for infection by several pathogens, and particularly vector-borne viruses [66]. Unfortunately, data on foreign-born residents are provided by ISTAT as an aggregate information. Consequently, we cannot rule out that our estimates may have been inflated by figures on foreign-born individuals born in areas not at risk for DENV infection. Similar criticisms should be moved to estimates on travel, as ISTAT does not provide accurate information on travel by single countries, being rather reported by large geographic areas, that may include regions with heterogenous risk for DENV infections.

Last but not least, our data lack significant information represented by the viral genotyping of the reported cases. According to available data, most cases described in available reports are DENV1 (45.6%), followed by DENV2 (26.7%), DENV3 (16.0%), and DENV4 (6.4%) [7,20,21,24,34,35,67]. As serotypes are increasingly associated with specific clinical features and risk of severe disease [68,69], with DENV2 more often resulting in DHF and DSS, the relatively lower share of DENV2 compared to the milder DENV1, DENV3 and DENV4 may represent a further clue of the extensive underdiagnosis of dengue in travelers, whose implication in the risk for the eventual endemization of this disorder are implicit.

## 5. Conclusions 

In conclusion, our study has characterized DENV infections in Italy as substantially clustered in international travelers. As a consequence, the main drivers of the epidemiology of DENV infections in Italy are represented by global epidemiology of the pathogen, particularly in endemic areas. The reported cases remain relatively rare, but a significant under-reporting of total cases cannot be ruled out owing to the clinical characteristics of DENV infection. Nevertheless, during the assessed timeframe 2010–2021, first autochthonous cases have been reported: due to the favorable settings offered by Italian climate, and the availability of a competent mosquito vector, without specifically targeted interventions it is likely that DENV could achieve an endemic status within the coming decades, eventually following the footsteps of other arboviruses (e.g., WNV). Accurate surveillance for DENV infection is therefore urgently needed.

## Figures and Tables

**Table 1:** Summary of notified cases of dengue in Italy (2010–2021).

Variable	No./1043, %
Gender	
Males	595, 57.0%
Females	444, 42.6%
N.A.	4, 0.4%
Age Group	
0 to 14	66, 6.3%
15 to 24	114, 11.0%
25 to 44	538, 51.6%
45 to 64	256, 24.6%
65 or more	50, 4.8%
N.A.	19, 1.8%
Geographic Area of Origin	
North-Western Italy	302, 29.0%
North-Eastern Italy	431, 41.3%
Central Italy	269, 25.9%
Southern Italy	27, 2.5%
Islands	14, 1.3%
Reporting year	
2010–2014	398, 38.2%
2015–2019	609, 58.4%
2020–2021	36, 3.4%
Reporting Month *	No./648, %
January	44, 6.8%
February	33, 5.1%
March	43, 6.6%
April	35, 5.4%
May	37, 5.7%
June	48, 7.4%
July	74, 11.4%
August	125, 19.3%
September	82, 12.7%
October	46, 7.1%
November	42, 6.5%
December	39, 6.0%
Source of the infection **	No./446, %
Autochtonous Cases	11, 2.5%
Central America	15, 3.4%
South America	14, 3.1%
Caribe	88, 19.7%
Africa and Middle East	30, 6.7%
Southeast Asia	116, 25.1%
Southern and Eastern Asia	121, 26.9%
Oceania	6, 1.3%
NA	50, 11.2%

* = data available for the time period January 2015–August 2021; ** = data available for the time period 2021–2018; 2016–2012; 2010.

**Table 2:** Notification rates for cases of dengue in Italy (2010–2021). Crude incidence rates (CIR) were estimated assuming the total Italian population as the reference. Age-adjusted standardized rates (ASR) with their respective 95% confidence intervals (95% CI) were calculated assuming standard European Population as the reference. Incidence in travelers was calculated through official data of Italian Institute for Statistics on Travelers in countries other than European Union (ISTAT).

Year	Incident Cases (No./1043, %)	Autochtonous Cases (No./Incident Cases, %)	CIR per 100,000 Persons (95% CI)	ASR per 100,000 Persons (95% CI)	Incidence per 100,000 Travellers (95% CI)
2010	51, 4.9%	-	0.085 (0.064; 0.087)	0.075 (0.070; 0.081)	0.325 (0.242; 0.427)
2011	47, 4.5%	-	0.060 (0.028; 0.091)	0.130 (0.124; 0.135)	0.360 (0.265; 0.479)
2012	79, 7.6%	-	0.074 (0.050; 0.098)	0.234 (0.225; 0.243)	0.541 (0.428; 0.673)
2013	142, 13.6%	-	0.131 (0.100; 0.162)	0.131 (0.125; 0.138)	1.248 (1.053; 1.470)
2014	79, 7.6%	-	0.111 (0.078; 0.145)	0.178 (0.173; 0.184)	0.857 (0.679; 1.067)
2015	116, 11.1%	-	0.141 (0.103; 0.179)	0.191 (0.184; 0.197)	1.147 (0.949; 1.374)
2016	106, 10.2%	-	0.186 (0.144; 0.227)	0.159 (0.154; 0.164)	1.137 (0.932; 1.374)
2017	94, 9.0%	-	0.111 (0.081; 0.141)	0.183 (0.177; 0.190)	0.808 (0.653; 0.987)
2018	108, 10.4%	-	0.205 (0.157; 0.253)	0.197 (0.185; 0.209)	0.997 (0.818; 1.202)
2019	185, 17.7%	-	0.227 (0.187; 0.267)	0.328 (0.314; 0.341)	1.514 (1.305; 1.746)
2020	32, 3.1%	11, 34.4%	0.032 (0.001; 0.064)	0.051 (0.048; 0.054)	0.723 (0.448; 1.103)
2021	4, 0.4%	-	0.011 (0.001; 0.015)	0.008 (0.004; 0.011)	-
POOLED	1043, 100%	11, 100%	0.114 (0.071; 0.183)	0.155 (0.105; 0.206)	0.796 (0.599; 1.057)

**Table 3:** Distribution of Italian cases of dengue by geographic area of residence and sex. A total of 1043 out cases were included in the analyses.

Variable	Risk Ratio (95% Confidence Interval)
Gender	
Males	REFERENCE
Females	0.704 (0.622; 0.796)
Age group	
0 to 14	0.238 (0.181; 0.302)
15 to 24	0.589 (0.481; 0.720)
25 to 44	REFERENCE
45 to 64	0.430 (0.371; 0.499)
65 or more	0.110 (0.082; 0.147)
Geographic Area of Origin	
North-Western Italy	REFERENCE
North-Eastern Italy	1.968 (1.699; 2.280)
Central Italy	1.205 (1.020; 1.417)
Southern Italy	0.102 (0.069; 0.152)
Islands	0.111 (0.065; 0.191)
Reporting year, Travellers	
2010–2014	REFERENCE
2015–2019	1.808 (1.594; 2.051)
2020–2021	1.771 (1.238; 2.534)
Reporting year, Residents	
2010–2014	REFERENCE
2015–2019	1.552 (1.352; 1.782)
2020–2021	0.487 (0.339; 0.699)

**Table 4:** Incidence rate ratios (IRR) for dengue cases in Italy (2010–2020) by demographics and main characteristics of the index cases. IRR were calculated by means of a Poisson logistic regression assuming as outcome variable the annual incident cases of dengue.

Variable	IRR (95% Confidence Interval)
Travelers per 100 inhabitants (+1.0%)	1.065 (1.036; 1.096)
Travels to South/Central America (+1000/year)	1.001 (0.001; 100.3)
Travels to Asia (+1000/year)	0.999 (0.001; 100.5)
Incidence in EU-EEA (+1000 cases/year)	1.323 (1.165; 1.481)
Population aged 25 to 44 (+1.0%)	1.622 (1.338; 1.968)
Foreign-born residents (+1.0%)	2.717 (1.555; 3.881)

## Data Availability

The data presented in this study are available on request from the corresponding author.

## Appendix A

**Table A1:** Cases of Dengue fever reported in Italy between 2010 and 2021.

	Region	YEAR												POOLED
		2010	2011	2012	2013	2014	2015	2016	2017	2018	2019	2020	2021	
North-Western Italy	Aosta Valley	-	-	-	-	-	-	0.785	-	-	-	-	-	0.065 (0.001; 0.194)
	Piedmont	0.114	0.023	0.113	0.271	0.090	0.271	0.318	0.387	0.206	0.254	-	-	0.171 (0.096; 0.245)
	Liguria	-	-	-	-	0.063	-	-	-	0.257	-	-	-	0.027 (0.001; 0.069)
	Lombardy	0.031	0.031	0.255	0.425	0.130	0.200	0.130	0.200	0.130	0.479	0.139	-	0.179 (0.095; 0.263)
North-Eastern Italy	AP Trento	-	-	-	0.187	-	0.186	0.372	0.371	0.926	0.368	-	-	0.201 (0.043; 0.359)
	AP Bolzano	-	-	-	-	0.582	0.771	0.192	0.191	0.758	0.754	-	-	0.271 (0.078; 0.463)
	Veneto	0.350	0.225	0.246	0.347	0.223	0.284	0.366	0.346	0.449	0.532	0.389	0.041	0.317 (0.245; 0.388)
	Friuli Venezia Giulia	0.082	0.362	-	-	-	-	0.164	-	0.329	0.413	-	-	0.109 (0.020; 0.199)
	Emilia Romagna	0.438	0.366	0.250	0.544	0.382	0.382	0.540	0.315	0.292	0.830	0.134	0.022	0.375 (0.257; 0.492)
Central Italy	Tuscany	0.054	0.134	0.268	0.401	0.133	0.453	0.240	0.294	0.268	0.216	-	-	0.205 (0.123; 0.288)
	Umbria	-	-	0.112	-	0.112	-	-	-	0.113	-	-	-	0.028 (0.001; 0.057)
	Marche	-	-	0.065	-	0.193	-	0.324	-	0.065	0.132	-	-	0.065 (0.006; 0.123)
	Latium	0.018	0.072	0.250	0.440	0.290	0.339	0.401	0.153	0.322	0.641	0.017	-	0.245 (0.132; 0.358)
Southern Italy	Abruzzo	-	-	-	-	0.075	-	-	-	-	-	-	-	0.006 (0.001; 0.018)
	Campania	-	-	-	-	-	0.017	0.017	-	-	-	-	-	0.003 (0.001; 0.007)
	Molise	-	-	-	-	-	-	-	-	-	-	-	-	-
	Apulia	0.049	0.073	-	0.122	0.024	0.049	-	0.074	0.131	0.075	-	0.025	0.052 (0.027; 0.077)
	Basilicata	-	-	-	-	-	-	-	-	-	-	-		-
	Calabria	-	-	-	-	-	-	0.051	-	-	0.052	-	-	0.011 (0.001; 0.020)
Islands	Sicily	0.020	-	-	0.020	0.039	-	-	-	-	0.020	-	-	0.008 (0.001; 0.016)
	Sardinia	-	-	-	-	-	-	0.061	-	-	-	-	-	0.005 (0.001; 0.015)
	TOTAL	0.055	0.060	0.074	0.131	0.111	0.141	0.186	0.111	0.205	0.227	0.032	0.004	0.111 (0.072; 0.151)
